# Case Report: A rare form of congenital erythrocytosis due to *SLC30A10* biallelic variants—differential diagnosis and recommendation for biochemical and genetic screening

**DOI:** 10.3389/fped.2024.1319885

**Published:** 2024-01-12

**Authors:** Rosalinda Giannini, Emanuele Agolini, Giuseppe Palumbo, Antonio Novelli, Giacomo Garone, Melissa Grasso, Giovanna Stefania Colafati, Marta Matraxia, Eleonora Piccirilli, Annalisa Deodati, Giulia Ceglie

**Affiliations:** ^1^ Department of Biomedicine and Prevention, University of Rome Tor Vergata, Rome, Italy; ^2^ Laboratory of Medical Genetics, Bambino Gesù Children’s Hospital, IRCCS, Rome, Italy; ^3^ Department of Pediatric Hematology and Oncology, Bambino Gesù Children’s Hospital, IRCCS, Rome, Italy; ^4^ Department of Systems Medicine, University of Rome Tor Vergata, Rome, Italy; ^5^ Clinical and Experimental Neurology, Bambino Gesù Children’s Hospital, IRCCS, Rome, Italy; ^6^ Department of Neuroscience, Mental Health and Sensory Organs, Faculty of Medicine and Psychology, Sapienza University of Rome, Rome, Italy; ^7^ Neurological and Neurosurgical Diseases Research Unit, Bambino Gesù Children’s Hospital, IRCCS, Rome, Italy; ^8^ Imaging Department, Bambino Gesù Children’s Hospital, IRCCS, Rome, Italy; ^9^ Endocrinology and Diabetes Unit, Bambino Gesù Children’s Hospital, IRCCS, Rome, Italy; ^10^ PhD Program in Immunology, Molecular Medicine and Applied Biotechnology, University of Rome Tor Vergata, Rome, Italy

**Keywords:** congenital erythrocytosis, hypermanganesemia, *SLC30A10*, HMNDYT1, genetic testing

## Abstract

Congenital erythrocytosis recognizes heterogeneous genetic basis and despite the use of NGS technologies, more than 50% of cases are still classified as idiopathic. Herein, we describe the case of a 3-year-old boy with a rare metabolic disorder due to SLC30A10 bi-allelic mutations and characterized by hypermanganesemia, congenital erythrocytosis and neurodegeneration, also known as hypermanganesemia with dystonia 1 (HMNDYT1). The patient was treated with iron supplementation and chelation therapy with CaNa2EDTA, resulting in a significative reduction of blood manganese levels and erythrocytosis indexes. Although it couldn't be excluded that the patient's developmental impairment was part of the phenotypic spectrum of the disease, after three months from starting treatment no characteristic extrapyramidal sign was detectable. Our findings suggest the importance of assessing serum manganese levels in patients with unexplained polycythemia and increased liver enzymes. Moreover, we highlight the importance of extended genetic testing as a powerful diagnostic tool to uncover rare genetic forms of congenital erythrocytosis. In the described patient, identifying the molecular cause of erythrocytosis has proven essential for proper clinical management and access to therapeutic options.

## Introduction

Erythrocytosis is characterized by an increase of red blood cells mass above the reference range adjusted to age, sex and living altitude, which is reflected in an elevation of hematocrit (Hct) and hemoglobin (Hb) concentration (1). Besides idiopathic forms, erythrocytosis can be classified as either primary or secondary, based on pathophysiology and circulating erythropoietin (EPO) levels. Primary erythrocytosis exhibits an EPO-independent mechanism and is usually linked to a molecular defect intrinsic to the RBC or its precursors. In contrast, secondary forms are identified as EPO-dependent and erythrocytosis is the consequence of erythroid progenitors’ responsiveness to increased EPO secretion. In idiopathic erythrocytosis, which accounts for most cases, the etiology has yet to be determined and there is no well-established correlation with specific EPO levels (2).

Primary and secondary forms can be either congenital—usually linked to a genetic defect—or acquired (Table 1). The best-known form of primary congenital erythrocytosis is due to heterozygous pathogenic variants in *EPOR* gene, which encodes for EPO receptor and more recently *SH2B3* germline mutations have been implied as another possible cause (3). No definite treatment guidelines have been published for primary congenital erythrocytosis and while most individuals require no regular treatment, some undergo phlebotomy to treat hyperviscosity symptoms (4). Genetic causes of secondary congenital erythrocytosis are certainly wider, including both autosomal dominant and recessive forms. Here, we report on the case of a 3-year-old boy, presenting with erythrocytosis and growth delay, harboring a bi-allelic variant in *SLC30A10* gene which is responsible for a rare recessive syndromic form of erythrocytosis known as hypermanganesemia with dystonia 1 (HMNDYT1).

**Table 1:** Primary and secondary forms of erythrocytosis, congenital or acquired.

Primary erythrocytosis		Secondary erythrocytosis	
Congenital	Acquired	Congenital	Acquired
ECYT^a^ 1 (*EPOR* gene) *SH2B3* gene	Polycythemia vera (including *JAK2* mutations)	ECYT 2 (*VHL* gene) ECYT 3 (*EGLN1* gene) ECYT 4 (*EPAS1* gene) ECTY 5 (*EPO* gene) ECTY 6 (*HBB* gene) ECTY 7 (*HBA1* and *HBA2* genes) ECTY 8 (*BPGM* gene) *PIEZO1* gene *SLC30A10* gene	Smoking High altitude Autonomous EPO production: - Kidney: nephroblastoma, RCC^b^ - Liver: hepatoma, HCC^c^ - CNS^d^: cerebellar hemangioblastoma, meningioma - Endocrine tumor: Pheochromocytoma Systemic/local hypoxia due to: - Pulmonary - Renal - Cardiac - Hepatic diseases Drug associated: - Erythropoietin/androgen administration

^a^ erythrocytosis familial.

^b^ renal cell carcinoma.

^c^ hepatic cell carcinoma.

^d^ central nervous system.

## Results

### Case description

The patient is the only child of consanguineous parents native to Bangladesh, born at 36 + 6 weeks of gestation. Weight at birth was 1.740 kg (*Z*-score −2.81), length was 41 cm (*Z*-score −2.19), occipito-frontal circumference was 30 cm (Z-score −2.95) (5). Developmental motor milestones were reached according to age (sitting position at 7 months, autonomous walking at 14 months), while first words were pronounced around 18 months of age and language development was delayed. In addition, from the age of two, parents noticed poor interaction with peers and poor non-verbal communication. The patient was initially evaluated at the age of 12 months for poor weight gain in the context of late preterm birth and SGA. Routinary blood tests showed a slight increase of RBC [5.300.000/mm^3^, upper reference limit (URL) 5.000.000/mm^3^] and AST activity (53 U/L, URL 40 U/L). Screening for celiac disease, TSH and IGF1 were normal while total IgE levels were increased (299 kU/L, URL 40 kU/L) with positivity for specific IgE against milk, alpha and beta lactoglobulin, for which a two-month cow's milk protein-free diet was started. On follow up exams, a trend towards progressive increase of erythocytosis was evident, and the patient was admitted to the hospital for further investigations. On admission, complete blood count showed: RBC 7.850.000/mm^3^, Hct 65.2%, Hb 19.8 g/dl, platelets 170.000/mm^3^ and reticulocyte 144.000/µl (2.2%). Hb electrophoresis and serum EPO levels were normal (6.63 mu/ml, normal range: 4.3–29 mu/ml). Polycythemia Vera and other forms of myeloproliferative neoplasms were excluded through molecular testing for *JAK2* -V617F mutation and *BCR/ABL* fusion gene on peripheral blood. Echocardiogram and complete abdomen ultrasound showed no anomalies. After initial treatment, the patient underwent phlebotomies in aliquots of 6 ml/Kg at rate of approximately 1 procedure per month. To investigate different congenital forms of polycythemia a clinical exome sequencing, containing more than 8,500 genes, including the ones involved in polycythemia (*EPOR, SH2B3, EGLN1, EPAS1, EPO, JAK2, PIEZO1, SLC30A10* and *VHL*), was performed. Data analysis did not detect any variant in primary erythrocytosis genes but revealed the homozygous novel missense variant NM_018713.3:c.392T>G (p.Leu131Arg) in *SLC30A10* gene (ZINC TRANSPORTER 10; ZNT10), associated with HMNDYT1 and initially classified as a variant of uncertain significance according to the ACMG criteria (see Supplementary Materials).

**Figure 1:**
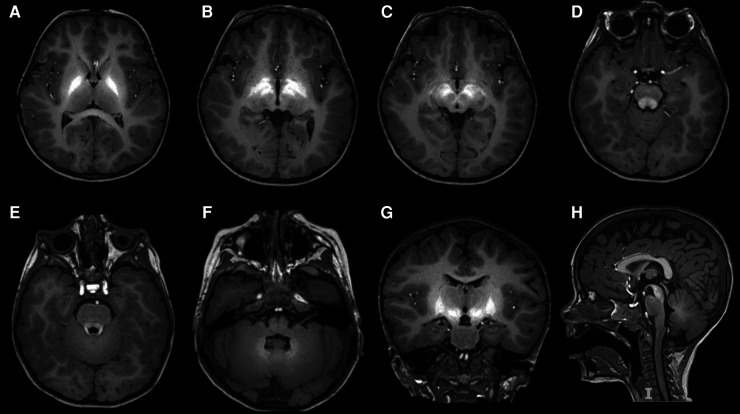
Bilateral and symmetrical hyperintensity in T1-weighted imaging involving globi pallidi (**A,B,G**), subthalamic nuclei (**B,G**), substantia nigra (**C,H**), pontine tegmentum (**D**), superior cerebellar peduncles (**E**), dentate nuclei (**F**), and pituitary gland (**E,G**), reflecting manganese accumulation.

To assess our patient's variant pathogenicity, serum Mn levels were tested, confirming a condition of hypermanganesemia (7.00  μg/L; URL 3.00 μg/L). Brain MRI showed bilateral and symmetrical T1 signal hyperintensity of globi pallidi, subthalamic nuclei, substantia nigra, pontine tegmentum, superior cerebellar peduncles, dentate nuclei and anterior pituitary gland, highly suggestive of manganese accumulation (Figure 1). Brain MRI findings along with erythrocytosis and hypermanganesemia confirmed the diagnosis of HMNDYT1, and the c.392T>G (p.Leu131Arg) variant in *SLC30A10* was re-classified as likely pathogenic. Hepatic ultrasound was normal, and no extrapyramidal sign was detected at neurological examination. A standardized neuropsychological evaluation showed a non-verbal IQ within normal range (IQ 87, Leiter 3 scale), with a poor adaptive functioning (Vineland-II Adaptive Behavior composite score 47, below the first centile for age). At 3 years of age, language is limited to two words, pointing gesture, pretend and symbolic play are absent, and eye contact is poorly modulated.

The patient was treated with iron supplementation and chelation therapy with CaNa2EDTA (20 mg/kg). Manganese levels were assessed before and after chelation therapy, showing a significative reduction from 19,80 μg/L–7,41 μg/L. Complete blood count showed a reduction of erythrocytosis indexes (RBC 5.840.000/mm^3^, Hct 45.9%, Hb 14.3 g/dl). After three months from starting treatment, our patient has not developed extrapyramidal signs. However, it remains unclear if his developmental impairment with language delay and autistic traits are part of the phenotypic spectrum of the disorder (although not reported yet) or an unrelated comorbidity.

## Discussion

HMNDYT1 is a form of hypermanganesemia presenting with parkinsonism, dystonia, polycythemia and chronic liver disease, associated with biallelic pathogenic mutations in *SLC30A10* gene. *SLC30A10* encodes a manganese (Mn) transporter, expressed in different tissues and responsible for Mn efflux from the Cytosol. Alteration of manganese metabolism leads to hypermanganesemia and to the accumulation of manganese in the liver, muscles and brain, particularly in the basal ganglia (6). HMNDYT1 usually presents with neurologic symptoms that can appear from early childhood to adulthood. In the childhood-onset form, neurologic involvement becomes apparent between two and fifteen years of age primarily as extrapyramidal signs including four-limbs dystonia, leading to a characteristic high-stepping gait (“cock-walk gait”), dysarthria, bradykinesia, and rigidity (7). In a few patients, onset can occur in adulthood as akinetic-rigid parkinsonism unresponsive to L-dopa treatment (6). Another complication due to severely defective manganese excretion is chronic liver disease, which shows a wide phenotypic variability, ranging from elevated liver enzymes and mild hepatomegaly to hepatic cirrhosis (7). Polycythemia is a constant feature of HMNDYT1 and usually precedes neurological dysfunction. The most accredited mechanism underlying erythrocytosis is manganese-induced EPO production, as supported by increased serum EPO levels in some patients (8, 7). Mimicking a condition of hypoxia, manganese and other transition metals have been suggested to influence EPO gene expression through the regulation of specific transcription factors (8).

In our case, clinical presentation consisted in polycythemia with iron deficiency and normal serum EPO levels, combined with failure to thrive and slightly increased serum AST activity (without other signs of liver dysfunction), in absence of movement disorders. This finding suggests that serum manganese levels should be assessed in patients with unexplained polycythemia and iron deficiency, even in absence of overt neurological dysfunction or EPO overproduction, and especially in case of abnormal liver tests or poor growth. This approach would probably translate into an earlier diagnosis of congenital errors of manganese metabolism, possibly preventing the occurrence of neurological symptoms.

Even though there is still no standardized treatment protocol for HMNDYT1, the primary treatment for this condition is chelation along with iron therapy. Studies showed that progression of manganese accumulation can be prevented through chelation therapy with CaNa2 EDTA, leading to an improvement of neurological symptoms (9). On the other hand, iron acts as a competitive inhibitor of intestinal manganese uptake and iron supplementation despite normal serum iron levels has proven to be helpful in reducing blood manganese concentration and normalizing erythrocyte count (7). In our case, chelation therapy was useful in managing polycythemia, and no extrapyramidal sign has emerged so far.

In conclusion, rare forms of congenital erythrocytosis for which a specific treatment option is available could benefit greatly from an early diagnosis. In young patients presenting with idiopathic isolated erythrocytosis (especially if a family history of consanguinity is present) congenital forms should always be considered and ruled out through specific biochemical and genetic testing (10). The use of NGS technology to diagnose infrequent forms of genetic determined erythrocytosis has proven to be extremely useful in selected patients, identifying new genes and improving the diagnostic rate up to 45.6% (11). Considering a cost-effective approach, an enlarged multigene panel for congenital erythrocytosis should include genes causing both primary and secondary forms of erythrocytosis (especially treatable causes such as inborn errors of manganese metabolism), to improve their diagnostic yield and disease-modifying potential.

## Data Availability

The original contributions presented in the study are included in the article/Supplementary Material, further inquiries can be directed to the corresponding author/s.

## Supplementary material

Supplementary Figure 1 Trend of hematocrit and manganesemia before and after chelation therapy. Orange and blue lines represent the values of hematocrit and manganesemia respectively. The dark blue horizontal arrow corresponds to given iron supplementation, while vertical green arrows represent phlebotomy treatment (6 ml/Kg) and vertical purple arrows indicate the first and second administration of chelation therapy with CaNa2 EDTA (20 mg/kg). Even though our patient underwent only two chelation cycles with CaNa2 EDTA, consistently with reference literature, oral iron supplementation and chelation therapy combined appear to stabilize and decrease Mn blood levels, even if still above the upper reference limit. As reported by Gulab and colleagues in 2017 (doi: 10.1055/s-0037-1608778) and Jagadish and colleagues in 2021 (doi: 10.1016/j.ebr.2021.100505), it is not unusual for Mn levels to remain mildly to moderately elevated even after chelation therapy.

## References

[1] Cakmak HM Kartal O Kocaaga A Bildirici Y. Diagnosis and genetic analysis of polycythemia in children and a novel EPAS1 gene mutation. PEDN. (2022) 63(6):613–7. 10.1016/j.pedneo.2022.06.006 36002380

[2] Bento C. Genetic basis of congenital erythrocytosis. Int J Lab Hematol. (2018) 40(S1):62–7. 10.1111/ijlh.12828 29741264

[3] Oh ST Zahn JM Jones CD Zhang B Loh ML Kantarjian H Identification of novel LNK mutations in patients with chronic myeloproliferative neoplasms and related disorders. Blood. (2010) 116(21):315. 10.1182/blood.V116.21.315.315

[4] McMullin MF. Congenital erythrocytosis. Int J Lab Hematol. (2016) 38(1):59–65. 10.1111/ijlh.12506 27161533

[5] Fenton TR Kim JH. A systematic review and meta-analysis to revise the fenton growth chart for preterm infants. BMC Pediatr. (2013) 13:59. 10.1186/1471-2431-13-59 23601190 PMC3637477

[6] Quadri M Federico A Zhao T Breedveld GJ Battisti C Delnooz C Mutations in SLC30A10 cause parkinsonism and dystonia with hypermanganesemia, polycythemia, and chronic liver disease. Am J Hum Genet. (2012) 90(3):467–77. 10.1016/j.ajhg.2012.01.017 22341971 PMC3309204

[7] Tuschl K Clayton PT Gospe SM Jr Gulab S Ibrahim S Singhi P Syndrome of hepatic cirrhosis, dystonia, polycythemia, and hypermanganesemia caused by mutations in SLC30A10, a manganese transporter in man. Am J Hum Genet. (2012) 90(3):457–66. 10.1016/j.ajhg.2012.01.018 22341972 PMC3309187

[8] Hebert BL Bunn HF. Regulation of the erythropoietin gene. Blood. (1999) 94(6):1864–77. 10.1182/blood.V94.6.1864 10477715

[9] Di Toro Mammarella L Mignarri A Battisti C Monti L Bonifati V Rasi F. Two-year follow-up after chelating therapy in a patient with adult-onset parkinsonism and hypermanganesaemia due to SLC30A10 mutations. J Neurol. (2014) 261:227–8. 10.1007/s00415-013-7187-5 24276520

[10] Jeevasankar M Agarwal R Chawla D Vinod KP Ashok KD. Polycythemia in the newborn. Indian J Pediatr. (2008) 75:68–72. 10.1007/s12098-008-0010-0 18245939

[11] Tomc J Debeljak N. Molecular pathways involved in the development of congenital erythrocytosis. Genes (Basel). (2021) 12(8):1150. 10.3390/genes12081150 34440324 PMC8391844

