# Supplementary material for: Case Report: A rare form of congenital erythrocytosis due to SLC30A10 biallelic variants—differential diagnosis and recommendation for biochemical and genetic screening
Source: Front Pediatr. 2024 Jan 12;12:1319885. doi: 10.3389/fped.2024.1319885 (PMC10811125; doi:10.3389/fped.2024.1319885)
Supplement: Supplementary file 1 [file Datasheet1.pdf]

**Supplementary information: Genetic studies**

Genetic analyses were conducted in accordance with the Helsinki Declaration. After obtaining informed consent from the patient's parents for the genetic testing, enrichment and parallel sequencing were performed on DNA from peripheral leukocytes, utilizing a custom clinical exome panel including *EPOR* and *SH2B3* genes, involved in primary forms of erythrocytosis and *EGLN1, EPAS1, EPO, JAK2, PIEZO1, SLC30A10* and *VHL* genes, associated with secondary erythrocytosis. Library preparation was carried out by using the Twist enrichment kit, according to the manufacture's protocol (Twist Bioscience, South San Francisco, CA), and sequenced on a NovaSeq 6000 (Illumina, Inc., San Diego, CA) platform. The BaseSpace pipeline (Illumina, https://basespace.illumina.com/) and the Juliaomix (GenomeUp, https://www.juliaomix.com) were used for germline and somatic variant calling and annotating variants, respectively. Sequencing data were aligned to the hg19 human reference genome. Exon coverage for erythrocytosis genes was 100%.

Data analysis revealed the homozygous novel variant NM_018713.3:c.392T>G (p.Leu131Arg) in *SLC30A10* gene (ZINC TRANSPORTER 10; ZNT10), associated with HMNDYT1. This missense change is non present in GnomAD database, is localized in the cation efflux domain, is predicted as deleterious and probably damaging by SIFT, Mutation Taster and PolyPhen 2.0, has a CADD score of 27 and was initially classified as variant of uncertain significance according to the ACMG criteria (PP3, PM1 and PM2). After the clinical diagnosis of hypermanganesemia with brain manganese accumulation, the criteria PP4 was added and the variant has been reclassified as likely pathogenic.
