# Supplementary figures and images for: Case Report: A rare form of congenital erythrocytosis due to SLC30A10 biallelic variants—differential diagnosis and recommendation for biochemical and genetic screening
Source: Front Pediatr. 2024 Jan 12;12:1319885. doi: 10.3389/fped.2024.1319885 (PMC10811125; doi:10.3389/fped.2024.1319885)

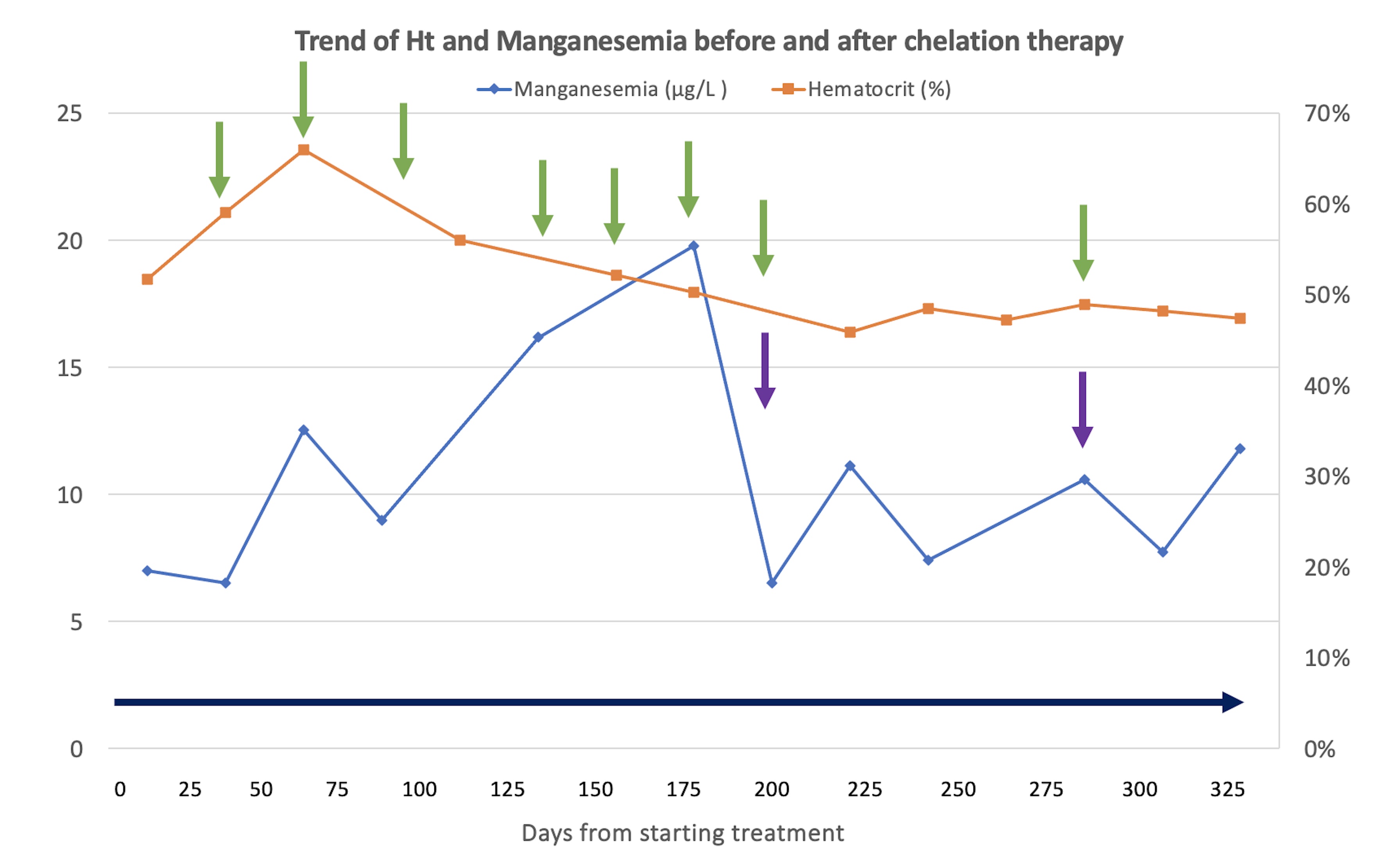

Supplement: Supplementary Figure 1 — Trend of hematocrit and manganesemia before and after chelation therapy. Orange and blue lines represent the values of hematocrit and manganesemia respectively. The dark blue horizontal arrow corresponds to given iron supplementation, while vertical green arrows represent phlebotomy treatment (6 ml/Kg) and vertical purple arrows indicate the first and second administration of chelation therapy with CaNa2 EDTA (20 mg/kg). Even though our patient underwent only two chelation cycles with CaNa2 EDTA, consistently with reference literature, oral iron supplementation and chelation therapy combined appear to stabilize and decrease Mn blood levels, even if still above the upper reference limit. As reported by Gulab and colleagues in 2017 (doi: 10.1055/s-0037-1608778) and Jagadish and colleagues in 2021 (doi: 10.1016/j.ebr.2021.100505), it is not unusual for Mn levels to remain mildly to moderately elevated even after chelation therapy. [file Image1.jpeg]
